# Recurrence rate and mesh bulging are reduced with primary fascial closure in ventral hernia repair: the PROSECO randomized clinical trial

**DOI:** 10.1093/bjs/znaf169

**Published:** 2025-09-02

**Authors:** Mikael Lindmark, Jael Tall, Bahman Darkahi, Johanna Österberg, Karin Strigård, Anders Thorell, Ulf Gunnarsson

**Affiliations:** Department of Diagnostic and Intervention, Umeå University, Umeå, Sweden; Skellefteå Research Unit, Skellefteå, Sweden; Department of Clinical Sciences, Danderyd Hospital, Karolinska Institute, Stockholm, Sweden; Department of Surgery and Anaesthesia, Ersta Hospital, Stockholm, Sweden; Department of Surgery, Enköping Hospital, Enköping, Sweden; Department of Surgery, Mora Hospital, Mora, Sweden; Department of Clinical Science and Education, Södersjukhuset, Karolinska Institute, Stockholm, Sweden; Department of Diagnostic and Intervention, Umeå University, Umeå, Sweden; Skellefteå Research Unit, Skellefteå, Sweden; Department of Clinical Sciences, Danderyd Hospital, Karolinska Institute, Stockholm, Sweden; Department of Surgery and Anaesthesia, Ersta Hospital, Stockholm, Sweden; Department of Diagnostic and Intervention, Umeå University, Umeå, Sweden

## Abstract

**Background:**

Laparoscopic intraperitoneal onlay mesh repair using a bridging technique has shown high rates of hernia site complications. Primary fascial closure before mesh placement has been utilized to address this. This randomized, parallel, double-blind, multicentre controlled trial investigated whether primary fascial closure reduces hernia site complications.

**Methods:**

Adults undergoing laparoscopic intraperitoneal onlay mesh repair for a midline hernia were randomized to primary fascial closure or bridging. Clinical assessment and the Ventral Hernia Pain Questionnaire were completed preoperatively and at 3 and 12 months post-surgery. CT scans were performed pre- and 12 months post-surgery. It was hypothesized that non-resorbable suture closure would reduce complication rates from 30% to 13% at 12 months, requiring 180 patients for 80% power and 95% significance.

**Results:**

One hundred and ninety-two patients were randomized (97 closure, 95 bridging), with 173 (90%) completing 1-year follow-up. At 12 months, overall hernia site complication rates showed no significant difference clinically (18% *versus* 20%, *P* = 0.85) or on CT (25% *versus* 28%, *P* = 0.50). However, recurrence and mesh bulging were significantly lower with fascial closure (4% *versus* 20%, *P* = 0.006). This group also reported significantly less pain at 12 months.

**Conclusion:**

Although there was no difference in the primary endpoint, fascial closure resulted in significantly lower rates of recurrence and mesh bulging, along with reduced postoperative pain. These findings suggest that primary fascial closure should be recommended alongside intraperitoneal onlay mesh repair in midline hernias.

**Trial Registration:**

The trial was registered at the ISRCTN at the start of the trial (ISRCTN51495042)

## Teaser

This study investigated whether fascial closure during laparoscopic ventral hernia repair reduces hernia site complications. The findings showed a statistically significant decrease in the combined rate of recurrence and mesh bulging with fascial closure. This, along with less postoperative pain, suggests primary fascial closure is advisable for intraperitoneal onlay mesh repair.

## Introduction

Ventral hernia repair is a frequently performed surgical procedure; however, the optimal surgical technique is still a matter of debate. High-quality data on key outcome variables are limited for both primary and incisional hernia repair^1,2^. Minimally invasive techniques have been shown to reduce surgical site complications compared with open surgery^3^, although no differences in outcomes such as recurrence, pain, reoperation, and mortality rate have been observed^3^.

The IPOM technique (laparoscopic intraperitoneal onlay mesh repair) was introduced in the 1990s as a bridging repair, in which the mesh overlaps the hernia defect with no attempt at closure^4^. One drawback of this approach is that it does not restore the integrity or function of the abdominal wall. In the repair of midline hernia defects with fascial closure, approximation of the rectus muscles restores the continuity of the abdominal wall muscle layer and potentially improves abdominal wall function^5,6^. Laparoscopic IPOM without closure of the hernia defect has been reported to result in only modest improvements in functional outcomes and patient satisfaction post-suregery^5,7^. In addition, mesh bulging, defined as pseudo-herniation of the mesh into the defect, and seroma formation are common complications of the bridging technique.

Guidelines from the European Hernia Society, American Hernia Society, and the International Endo-Hernia Society recommend fascial closure prior to mesh placement. This was based on two systematic reviews, including observational studies that have shown lower rates of recurrence and seroma^8,9^. Several randomized trials have been conducted in recent years. Bernardi *et al.* performed a study on 129 patients with quality-of-life as the primary outcome, showing this to be higher after fascial closure.^6^ However, no differences in rates of surgical site complications, recurrence, or mesh bulging were reported. In a meta-analysis based on pooled data from three studies, no difference was observed between fascial closure and non-closure with respect to rates of recurrence, seroma formation, mesh bulging, or severe adverse events. However, none of the studies in the meta-analysis had the power to detect clinically relevant differences^10^.

This study aimed to evaluate whether fascial closure prior to mesh placement reduces hernia site complications 1 year after IPOM.

## Methods

### Study design

This Prospective RandOmised Study of Endoscopic fascia Closure and long-term Outcome (PROSECO) was a randomized, parallel, multicentre, double-blinded controlled trial. The trial was registered at the ISRCTN at the start of the trial (ISRCTN51495042). The study is reported in line with the CONSORT checklist and was approved by the Regional Ethics Board of Umeå University (Sweden), 18/06/2015ref: 2015-215-32M. Recruitment took place from November 2015 to September 2020.

### Participants

The inclusion and exclusion criteria are presented in *Table 1*. Multiple abdominal wall defects were permitted, and where present were summarized according to the guidelines of the European Hernia Society^11^. The defect area was calculated by multiplying the transverse and longitudinal lengths.

**Table 1:** Inclusion and exclusion criteria

Inclusion criteria	Exclusion criteria
Midline hernia	Recurrent incisional hernia
Primary or incisional hernia with a transverse diameter ≥2 to ≤8 cm	Patient not eligible for laparoscopic repair
Age >18 years	Prosthetic mesh in the abdominal wall after previous surgery
Able to understand verbal and written information in Swedish	Ostomy hernia

Patients were recruited at the outpatient clinic and provided with verbal and written information before consenting to participate in the study. Written consent and case report forms were acquired and stored by a research coordinator in accordance with GDPR requirements. Baseline characteristics and preoperative Ventral Hernia Pain Questionnaire (VHPQ) results were also collected.

### Randomization and allocation concealment

Randomization was performed after adhesiolysis (if required), repositioning of the hernia sac, measurement of the size(s) of the defect(s), and confirmation that all criteria were fulfilled and both techniques were applicable. Outcome assessors and patients were blinded to the allocation. The randomization sequence was computer-generated using a random number technique. Allocation to the treatment arm was provided online using a secure web-based application. Participants were randomly assigned to either the control or experimental (fascial defect closure) arm on a 1:1 basis. Permuted blocks were used and randomization was stratified by centre. The sizes of the permuted blocks varied randomly within predefined concealed limits. Permuted block sizes were not disclosed to the participating clinics. The process of randomization and allocation was completed on the study homepage, with coding performed by a system developer independent of the study recruitment, conduct, and analysis. The centre-specific allocation sequences were stored on a secure server accessible only to the system developer at the Information and Communication Technology Services and System Development, Umeå University.

### Surgical procedure

All interventions were standardized according to a predefined study protocol. The only difference between the two treatment arms was the closure of the hernia defect before mesh placement. The first author (M.L.) visited all participating centres prior to enrolment to confirm that the procedures were performed according to the protocol. All operating surgeons were experienced in intracorporeal fascial closure. Surgery was performed under general anaesthesia with a single dose of antibiotics (Eusaprim^®^ 160 mg + 800 mg and Metronidazol^®^ 1500 mg) administered preoperatively. Any adhesiolysis required was performed mainly by sharp cold dissection, and the abdominal wall was cleared from any fatty tissue to facilitate mesh ingrowth and fixation. The defect size was measured with an intra-abdominal pressure of 8 mmHg. The prosthetic mesh used in this study was Symbotex^®^ (Medtronic, Minneapolis, MN, USA) which was fixed with a resorbable tacker Securestrap^®^ (SS; Ethicon US, LLC, Ethicon, Inc., Somerville, NJ, USA) in a double-crown manner^12^. Temporary trans-fascial sutures were allowed during mesh positioning, but not as permanent fixation. In patients randomized to undergo fascial closure, a running suture of self-retaining non-resorbable V-loc^®^ (2.0 or 0) (Medtronic, Minneapolis, MN, USA) was used. The minimum mesh overlap of the fascia was set at 50 mm in any direction regardless of whether the hernia defect was closed or not. Port sites >5 mm were closed with slowly resorbable polydioxiane (PDS 2.0, (SS; Ethicon US, LLC, Ethicon, Inc., Somerville, NJ, USA)).

As the study was double-blinded, the operating surgeon did not attend study-specific or clinical visits until the predetermined 12-month follow-up was completed. Details of the operative management of the hernia defect were not entered in the patients’ medical records, thus the patient and surgeon who performed follow-up were both blinded to the treatment allocation. Follow-up clinical visits and VHPQ were performed at 3 and 12 months. A CT scan with Valsalva manoeuvre was performed at 12 months, and any pathological findings were validated by independent reviewers.

### Ventral hernia pain questionnaire

The VHPQ is a validated instrument used to assess pain associated with daily activities and patient satisfaction^13^. The first six questions measure pain intensity and duration, the next seven address pain during various daily activities such as walking, driving, and sitting, and the final seven questions assess patient satisfaction with results such as abdominal wall function and appearance.

### Outcomes

The primary endpoint was a composite measure of the hernia site complication rate within 12 months. Hernia-site complications were defined as either recurrence of the hernia, sustained and clinically significant seroma, superficial infection, and/or clinically significant mesh bulging. Bulging of the mesh was defined as protrusion of the mesh beyond the anterior plane of the anterior rectus fascia and through the hernia orifice with intact peripheral fixation. Endpoints were assessed at clinical visits and by CT scan. The main secondary clinical outcome was CT-verified recurrence and/or mesh bulging. Other secondary outcome measures included abdominal wall pain, as assessed by the VHPQ, surgical complications, and operation time.

### Sample size and statistics

Any of the hernia site complications defined above have been reported to occur in approximately 30% of patients operated without fascial closure^14^. The hypothesis was that the occurrence of hernia site complications after 12 months would be reduced to 13% if the hernia defect was closed with a running non-resorbable suture^15^. A Z-test determined that a total of 180 evaluable patients were needed to detect this difference with 80% power and 95% significance. In case of any protocol violation and/or dropout after randomization, three additional patients were included to maintain power. The patient data were registered in an Access^®^ database (Microsoft Corp.). Statistical analyses were performed using Statistica version 12 (StatSoft, Tulsa, OK, USA). The Mann–Whitney *U* test and Wilcoxon signed-rank test were used for ordinal scale variables. Fisher’s exact test and chi-square test were used for dichotomous scale variables. Statistical significance was defined as *P* ≤ 0.05. Absolute risk reduction (ARR) and number needed to treat (NNT) were used to enhance the interpretation and translate results into clinically relevant and easily understandable terms.

## Results

**Fig. 1:**
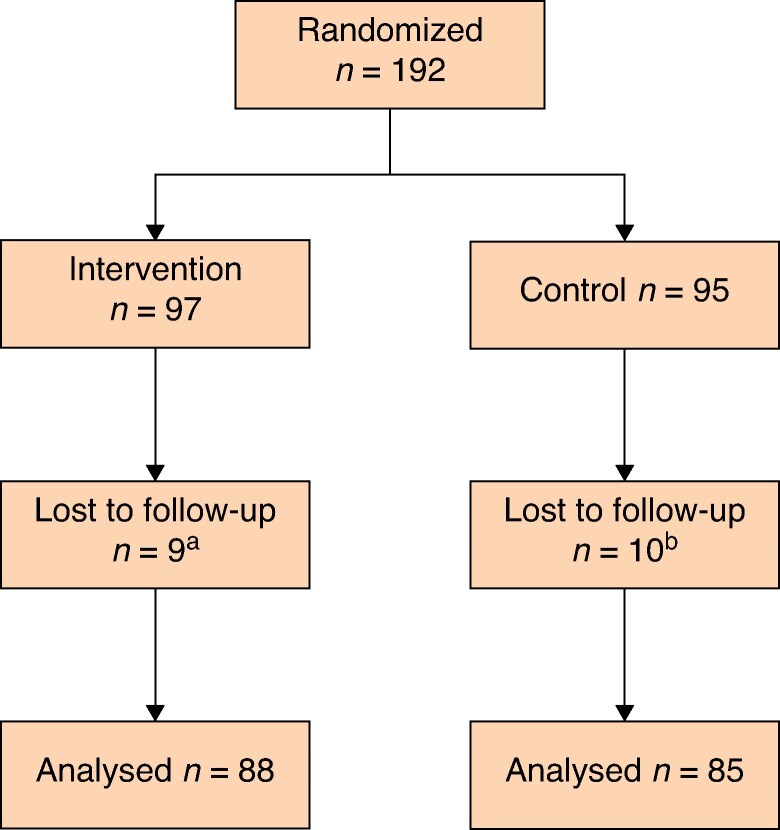
Flow chart a = no mesh inserted during operation (*n* = 1). Unable to reach (*n* = 4). Declined participation in follow-up (*n* = 3). Centre decided not to follow up due to complications (*n* = 1). b = Preoperative decision to perform open repair instead of laparoscopy (*n* = 1). Unable to reach (*n* = 5). Declined follow-up (*n* = 4)

A total of 192 patients were enrolled in the study, of which 97 were randomized to the fascial closure group and 95 to the non-closure group (*Fig. 1*). Four centres performed 186 procedures (97%), and the remaining six patients were operated at two centres that only participated at the beginning of the study.

There were no differences between the control and experimental groups in terms of baseline characteristics (*Table 2*). The number of primary and incisional hernias was comparable between the two treatment arms, with 37 incisional hernias in each group. The median operative time was 70 min for all patients (*Table 3*), and 8 min longer in the closure group (*P* = 0.02). The median transverse dimension of the hernias was 30 (21–79) mm, median hernia length was 30 (10–200) mm, giving a median hernia defect area of 9 cm^2^ (95% c.i. 2.5 to 15.8). Of the patients randomized to closure of the defect, 90 of 93 had complete closure. Patients with incomplete closure had a large incisional hernia with a transverse width of 50–79 mm. No patient was converted from laparoscopy to open surgery. The patients lost to follow-up are listed in the legend of *Fig. 1*.

**Table 2:** Baseline characteristics

Characteristics	Closure group (*n* = 97)	Non-closure group (*n* = 95)
Age (years)	57(12)	55(13)
**Sex**		
Male	58 (55)	59 (54)
Female	41 (45)	41 (46)
BMI, kg/m^2^	31(5)	32(6)
**ASA class**		
1	14 (18)	8 (10)
2	51 (66)	61 (75)
3	12 (16)	12 (15)
Smoking	5 (6.5)	6 (7)
**Primary hernia**		
M2	19 (20)	18 (20)
M3	33 (35)	32 (35)
**Incisional hernia**		
M1–M3	28 (30)	28 (31)
M4–M5	9 (10)	9 (10)

Age and BMI presented as mean(s.d.). Smoking presented as the actual number of patients that reported smoking. Hernias characterized according to the EHS midline hernia classification^11^, presented as the actual number within each group. Percentages are presented in brackets. Some primary hernia procedures did not report type of hernia hence percentages not reaching 100%.

**Table 3:** Details of surgery

Characteristics	Closure (*n* = 93)	Non-closure (*n* = 93)	*P*
Operation time (min)	75 (28–200)	67 (35–180)	0.02
Bleeding (ml)	5 (0–200)	3 (0–200)	0.5
Hernia above arcuate line (*N*)	73	73	1.0
Primary hernia (*N*)	50	49	0.88
Incisional hernia (*N*)	37	39	0.76
Hernia size transverse (mm)	30 (21–79)	30 (21–70)	0.77
Hernia size longitudinal (mm)	30 (10–200)	30 (15–150)	0.67
Needing two meshes (*N*)	7	2	0.09
Number of Secure straps® (median, range)	36 (12–60)	35 (20–60)	

186 complete forms from surgery available. Mann–Whitney *U* test analysis. Median value and range.

One patient in the closure group suffered mechanical intestinal obstruction in the early postoperative period which necessitated reoperation. The obstruction was caused by a loop of small bowel becoming trapped in an opening in the peritoneum created during dissection. During reoperation the mesh was removed and consequently the hernia recurred. Another patient in the closure group had an inadvertent colonic perforation that was not detected during index surgery. At reoperation the patient underwent formation of a colostomy, but at this time a duodenal injury was sustained. The centre that performed the operation decided to exclude the patient from the follow-up, and no results were available for further analysis.

At 12 months, no difference in overall hernia site complication rate was observed during clinical assessment (*P* = 0.85). Details of recurrence and complications are shown in *Table 4*. When analysing recurrence and mesh bulging combined, a significant difference was observed in favour of fascial closure (5% *versus* 20%, *P* = 0.006).

**Table 4:** Hernia site complications by clinical diagnosis and with CT scan evaluation at 12 months

Hernia site complication			*P*	ARR	NNT
**Clinical diagnosis, 12 months**	**Closure** **(*N* = 88)**	**Non-closure** **(*N* = 85)**			
Seroma	8 (9)	4 (5)			
Recurrence	3 (3)	2 (2)			
Infection	0 (0)	1 (1)			
Mesh bulging	5 (6)	10 (12)			
Total	16 (18)	17 (20)			
**CT scan evaluation, 12 months**	**Closure** **(*N* = 79)**	**Non-closure** **(*N* = 82)**			
Seroma	7 (9)	6 (7)			
Recurrence	2 (3)	6 (7)		5%	20
Infection	0 (0)	0 (0)			
Mesh bulging	2 (3)	10 (12)	0.02	10%	10
Total patients with hernia site complication (clinical and CT)	20 (25)	23 (28)	0.5		
Port site hernia	3 (4)	1 (1)			

The same patient could have several complications. Some complications were only seen on CT and not clinically. If the same complication was detected both on CT and clinically, it is only counted once. Some patients were only evaluated clinically, other patients by CT only. Percentages are presented in brackets. ARR = absolute risk reduction; NNT = numbers needed to treat.

Chronic pain, defined as a pain score of ≥3 (pain that cannot be ignored, affecting concentration and performance of daily activities), was evaluated before surgery and at the 12-month follow-up using the VHPQ (*Table 5*). There were no pre- or postoperative differences between the groups in the number of patients reporting ‘Pain right now <3’. However, at 12 months after surgery fewer patients in the closure group reported ‘Pain right now ≥3’ as well as ‘Pain last week ≥3’ compared to the non-closure group (2 *versus* 8, *P* = 0.05 and 1 *versus* 8, *P* = 0.016). None of the patients in the closure group and two patients in the non-closure group had pain that interfered with most daily activities. In a subgroup analysis a trend towards a higher prevalence of chronic pain (defined as a VHPQ score >3) among patients exhibiting mesh bulging (20%) compared to those without bulging (5%, *P* = 0.06) was identified. The ARR for chronic pain in patients without mesh bulging was 14%, with an NNT of seven.

**Table 5:** VHPQ pain score answers before surgery and at the 12-month follow-up

VHPQ pain score	Preoperative			Postoperative at 12 months			NNT	ARR
	Closure (*N* = 77)	Non-closure (*N* = 81)	*P*	Closure (*N* = 79)	Non-closure (*N* = 79)	*P*		
Pain right now ≤ 1	53	47	0.115	57	49	0.072		
Pain right now > 1 < 3	21	32	0.104	17	28	0.05	7	14%
Pain right now ≥ 3	33	44	0.149	2	8	0.05*	13	8%
Pain last week ≤ 1	33	23	0.057	49	48	0.87		
Pain last week > 1 < 3	39	52	0.058	24	29	0.540		
Pain last week ≥ 3	34	39	0.615	1	8	0.016*		
Difficulty rising from chair	14	15	0.829	1	5	0.209*		
Difficulty sitting	14	17	0.720	3	3	1.0*		
Difficulty standing	14	18	0.509	2	7	0.167*		
Difficulty climbing stairs	11	12	0.890	2	2	1.0*		
Difficulty driving a car	4	6	0.496*	1	1	1.0*		
Difficulty performing sports and physical activity	33	39	0.402	5	10	0.2775*		
Analgesics used past week	13	15	0.703	7	7	1.0*		

Chi-square used for all analyses except results marked with (*) which were analysed using Fisher’s exact test due to small sample size. Pain score 1 = pain that can easily be ignored. Pain score 3 = pain that cannot be ignored, and that affects concentration and performance of daily activities. ARR = absolute risk reduction; NNT = numbers needed to treat.

At 12 months, 68 of 77 patients (88%) in the closure group and 61 of 75 (81%) in the non-closure group were satisfied with abdominal wall function (*P* = 0.23), whereas 68 of 77 of patients in the closure group (88%) and 62 of 75 (83%) patients in the non-closure group were satisfied with aesthetic appearance (*P* = 0.32).

## Discussion

In this randomized, controlled, multicentre study, closure of the fascial defect in small- to medium-sized midline hernias did not reduce the primary endpoint: overall hernia site complication rates 1 year after laparoscopic IPOM with double crown fixation technique^1^. However, a post-hoc analysis specifically comparing the most clinically relevant outcome, recurrence and/or mesh bulging, detected a significant advantage in favour of fascia closure. Moreover, chronic pain was more common in the non-closure group.

This is the first trial to provide evidence that suturing the defect during ventral hernia repair reduces the rate of mesh bulging/hernia recurrence. Previous clinical trials and a recent meta-analysis failed to demonstrate corresponding results, probably owing to a lack of power^5,10,16,17^. In the present study, mesh bulging occurred in 12% if the fascia was not closed. This is similar to the findings from a meta-analysis of three randomized trials and 11 cohort studies that reported mesh bulging in 12% of patients whose fascial defect was left open^18^. Patients with mesh bulging often present with pain and discomfort, mimicking symptoms of true recurrence. From the patient’s perspective, bulging and recurrence might feel the same^19,20^. This is also supported by the finding in this study of a higher prevalence of pain in patients with bulging, suggesting that this may be an important variable associated with an increased risk of postoperative discomfort. The most important clinical difference between recurrence and mesh bulging is that patients with mesh bulging do not have the risk of incarceration^14,19^. If a patient has severe symptoms from mesh bulging, reoperation might be necessary^21^, but at the 1-year follow-up point no patient had been reoperated for bulging. Previous RCTs have evaluated the effect of defect closure using percutaneous trans-fascial sutures^5^ as well as an open hybrid approach^16^. In the present study, an intracorporeal suturing technique with a non-resorbable self-retaining suture was used to close the defect, although it is not certain that our results can be compared with those of studies using other closure techniques. The increase in operation time due to suturing in this study was 8 min, indicating that intracorporeal suturing with non-resorbable self-retaining sutures is not unreasonably difficult or time-consuming for defects ≤8 cm. Compared with other methods, intracorporeal suturing has the advantage that the skin barrier is not compromised. The study’s exclusion of hernias exceeding 8 cm limits the generalizability of fascial closure to larger defects. However, given that the majority of abdominal wall hernias present with a transverse diameter below 8 cm, the findings remain applicable to a substantial proportion of this patient population.

A report from the Danish Hernia Database investigated whether defect closure during laparoscopic IPOM reduces the reoperation rate for recurrence when compared with non-closure. The adjusted cumulative reoperation rate was significantly reduced with defect closure and permanent tacks (HR = 0.53, 95% c.i. = 0.28 to 0.999, *P* = 0.05)^22^. It is important to note that reoperation rate does not reflect recurrence rate and has been reported to represent only 20–25% of the true recurrence rate^23^.

In another Danish trial, 80 patients were recruited to investigate the effects of fascial closure on umbilical hernia repair. They found a higher recurrence rate in the no-closure group^24^; however, the primary endpoint was pain during physical activity and the study was not powered to assess hernia recurrence or seroma formation. This study reported a very high overall recurrence rate, which was likely caused by the use of an inferior mesh product which has subsequently been withdrawn from the market.

The risk factors for recurrence include surgeon-, patient-, and mesh-related variables. These include mesh pore size, mesh placement^25^, fixation method, co-morbidities, postoperative infection, and the surgeon’s annual hernia repair volume^26^. One could postulate that the increased area of the mesh adhering to the abdominal wall when closing the defect is beneficial for integration of the prosthetic material. Results from the 3-month follow-up of patients from the PROSECO study with the highest VHPQ scores prior to surgery showed the most pronounced postoperative improvement in VHPQ scores, regardless of treatment allocation^27^.

Restoring the abdominal wall anatomy and achieving tension-free repair are two cornerstones in hernia surgery that may counteract one another. There is a concern that suturing the hernia defect could increase tissue tension, thereby increasing the risk of chronic pain. In the present study, more pain was reported in the non-closure group at the 12-month follow-up, suggesting that anatomical restoration can decrease the risk of chronic pain. A reasonable explanation for the reduced pain and discomfort might be the prevention or reduction of mesh bulging and recurrence, as reported above. In cases of anatomically separate hernia defects, some patients received two distinct mesh prostheses rather than a single larger one. The utilizsation of two meshes could potentially result in a reduced overall quantity of prosthetic material implanted, although mesh size itself constitutes an independent risk factor for mesh-related complications^28^.

Despite being standard of care in ventral hernia repair for many years, laparoscopic IPOM is on a decline in parallel with an increase in the use of open sublay and new minimally invasive extraperitoneal techniques. Current trends in ventral hernia repair emphasize methods that avoid intraperitoneal mesh placement, such as the enhanced-view totally extra peritoneal (eTEP) and ventral transabdominal preperitoneal (TAPP) approaches. Given that defect closure is an inherent component of eTEP, our data may also be relevant to discussions concerning these alternative methods. Another reason for the decline of laparoscopic IPOM may be the hesitation of many surgeons to use the intraperitoneal mesh positioning and the assumed associated higher risk of mesh-related complications. However, there are no robust data to either confirm or refute the concerns of higher rates of adhesions and adhesion-related complications after ventral hernia surgery with intraperitoneal meshes^29^.

The inclusion of both primary and incisional hernias may be a limitation of this study; however, these were evenly distributed between the two groups. Compared with previous reports, the overall number of complications was lower in both arms of the study. A contributing factor to this might be that the size of the hernias in the study was relatively small^30^. Patient satisfaction regarding abdominal wall function and aesthetic appearance remained high (81–88%) at 12 months in both groups. Consequently, the observed disparities in mesh bulging and pain did not translate to a corresponding difference in self-reported function and aesthetic appearance. A similar finding with relatively high patient satisfaction, even in the presence of complications such as mesh bulging, was reported in a study of 210 patients^31^. Another limitation of our study was that the analysis of combined mesh bulging and recurrence rates was not explicitly specified in the original study protocol. However, given the well-documented impact of both conditions on patient well-being, pain, and quality of life, we believe that this post-hoc analysis is justified.

A strength of this study is the large number of patients included, with a very high follow-up rate (90%). The baseline characteristics were evenly distributed between the two groups, as expected in a well-designed RCT. Another strength is the pragmatic multicentre design, providing outcomes from several units, and a double-blinded design to prevent bias.

## Conclusion

This is the first study to provide high-quality evidence that closure of the hernia defect before mesh placement in laparoscopic IPOM reduces the rate of recurrence/mesh bulging. Furthermore, at the 1-year follow-up the closure group reported significantly less pain than the non-closure group. Taken together, these findings strongly support restoration of the abdominal wall using non-resorbable sutures prior to mesh placement in IPOM hernia repair.

## Data Availability

Data are available upon reasonable request. Deceased: Ulf Gunnarsson
